# Systematic Review of Assessment in General Chemistry: Trends, Digital Innovations, and Integration of Theory-Laboratory Course

**DOI:** 10.12688/f1000research.182947.1

**Published:** 2026-08-08

**Authors:** Oktariani Oktariani, Nahadi Nahadi, Heli Siti Halimatul Munawaroh, Lilit Rusyati, Andhika Baruri, Mellyzar Mellyzar, Desi Ariyanti Nabuasa, Almubarak Almubarak

**Affiliations:** 1 Science Education, Universitas Pendidikan Indonesia, Bandung, West Java, Indonesia; 2 Chemistry Education, Universitas Pendidikan Indonesia, Bandung, West Java, Indonesia

**Keywords:** assessment, general chemistry, literature review, PRISMA

## Abstract

**Background:**

Assessment in general chemistry plays an important role in supporting students’ conceptual understanding and laboratory competencies. However, studies that systematically analyze and synthesize research findings related to assessment practices in both theoretical and laboratory-based general chemistry learning remain limited. Previous studies also indicate inconsistencies in the integration of assessment approaches across theoretical and practical learning contexts. Therefore, a comprehensive systematic literature review is needed to identify current trends, gaps, and future directions in general chemistry assessment research.

**Methods:**

This study employed the Systematic Literature Review (SLR) method using the PRISMA approach. Articles were collected from the Scopus database covering publications from 2020 to 2024 (n = 30). The review process included identification, screening, eligibility assessment, and inclusion of relevant studies based on predetermined criteria. The selected articles were analyzed to examine assessment strategies, assessment focus, technological innovations, and research trends in general chemistry education.

**Results:**

The findings revealed several significant trends and gaps in the literature. Research on assessment in general chemistry is still predominantly focused on theoretical learning assessment rather than laboratory assessment or the integration of both domains. In addition, summative assessment practices remain underrepresented in the reviewed studies. The review also identified the growing implementation of technology-based assessment innovations, including digital question banks, machine learning-assisted assessment systems, and interactive assessment platforms. These technologies are increasingly used to enhance assessment effectiveness, accessibility, and student engagement.

**Conclusion:**

This review highlights the need for more balanced and integrated assessment approaches that combine theoretical and laboratory learning in general chemistry education. Future studies should further explore comprehensive assessment models that incorporate both formative and summative dimensions supported by technological innovations. The findings of this study provide evidence-based insights for educators, researchers, and policy makers in designing more innovative, relevant, and inclusive assessment practices to improve learning outcomes in general chemistry education.

## 1. Introduction

General chemistry lectures have an important role in providing the basics of essential scientific knowledge and skills needed to study other branches of chemistry. In practice, general chemistry lectures include theory and laboratory lectures which are carried out in an integrated or separate manner as each course. General chemistry theory lectures aim to build students’ conceptual and theoretical understanding of chemical principles, basic laws, and theories that are the basis for understanding chemical phenomena and creating a strong knowledge base that will support further learning in chemistry and related sciences. This approach emphasizes mastery of content, such as the concepts of molarity, stoichiometry, acid-base properties, thermodynamics, and chemical kinetics, which are necessary to develop analytical thinking skills and solve problems abstractly.

In contrast, general chemistry laboratory is designed to equip students with practical skills, including the ability to conduct experiments, analyze data, and interpret results empirically. Laboratory activity also aims to instill a deeper understanding through direct experience, allowing students to link the theory they have learned with its application in real situation ( Mardhiya & Laila, 2022; Veale et al., 2020). Laboratory activities in the form of laboratory are a core component because they provide opportunities for students to gain hands-on experience that supports understanding of theory while developing 21st century skills, such as collaboration, scientific communication, and data-based decision making ( Gao et al., 2021; Kolil et al., 2020; McKnelly et al., 2020; Singh et al., 2021). Thus, laboratory not only supports theoretical learning but also provides insight into the scientific method and research process in chemistry.

Integration between general chemistry theory and laboratory is often a challenge in designing chemical education curricula, considering that both have different but complementary objectives. The difference in focus in learning theory and laboratory certainly requires the formulation of special assessments in order to effectively measure the achievement of learning objectives in the lecture theory and laboratory of general chemistry ( Burrows et al., 2021; Veale et al., 2020).

In general chemistry courses, assessment serves to evaluate both conceptual knowledge and practical skills, especially in the laboratory context, where students must demonstrate competencies such as experimental design, data analysis, and problem solving. In addition, assessment is also needed to provide constructive feedback and encourage improved learning outcomes. Assessment in general chemistry has complexity characteristics that are different from other fields of science.

On the one hand, theoretical learning involves abstract concepts such as molecular structure and interactions, chemical bonding, reaction mechanisms, and thermodynamics that require deep cognitive engagement ( Ganajová et al., 2021). Assessments that previously focused only on concept understanding have shifted towards assessing 21st century skills such as critical thinking, creativity, collaboration, communication, problem solving, etc. ( Abaniel, 2017; Arsad et al., 2011; Peretz et al., 2023; Kelley et al., 2019). On the other hand, laboratory demands the application of these concepts through direct experimentation, which requires accuracy, analytical thinking, skills and collaboration. In addition, the number of concepts that must be learned by students sometimes makes the assessment ignore the depth of the concept and its validity ( Balabanoff et al., 2021). However, in its implementation, the assessment is still dominated by the assessment of students’ final reports, not based on performance during laboratory. Therefore, designing assessments that can effectively measure both dimensions is still a challenge ( Christenson & Walan, 2023). This challenge requires diverse and customized strategies to meet the needs.

Various assessment methods have been developed along with the transformation in the world of education. Conventional assessment methods such as written exams, laboratory reports and rubrics that have been the main pillars in assessing chemistry learning have begun to develop along with technological advances. Innovative tools such as virtual laboratories, gamified assessments, and Artificial Intelligence (AI)-based platforms have been introduced. These technologies enable interactive and personalized evaluation approaches and promise to increase student engagement and provide immediate feedback ( Lawrie, 2023; Schmid et al., 2020; Yakob et al., 2023). Nonetheless, traditional methods remain irreplaceable, especially in regions or institutions with limited access to technology. In well-resourced environments, technological advances offer opportunities for innovation, while in resource- constrained contexts, traditional methods remain the mainstay. This disparity highlights the need for strategies that can be adapted across different educational contexts.

Although there are many studies that discuss assessment strategies in education, studies that specifically focus on general chemistry lectures and laboratory practices are still relatively limited. Most of the approaches applied still rely on conventional summative assessments, such as tests and quizzes, which are often less effective in assessing students’ practical skills and conceptual understanding in depth. On the other hand, the application of formative assessment strategies, such as peer evaluation or providing direct feedback in the laboratory environment, has not received much attention in research.

Formative assessment is an important instrument that helps to improve the learning process and student understanding ( Ochsen et al., 2023). Formative assessment is a method of collecting data related to learning that is carried out throughout the learning process to provide feedback on students’ level of understanding so that learning can be adjusted to address gaps in students’ conceptual and procedural understanding ( Dalby & Swan, 2019; Kaushal Kumar & Spector, 2017). However, in classroom learning this assessment is often overlooked due to teachers’ different perceptions on how to implement formative assessment effectively and often face obstacles in doing so ( Abell & Sevian, 2021; Den Otter et al., 2022; Hagos & Andargie, 2024; Schafer & Yezierski, 2021).

The limited literature that systematically analyzes and summarizes various research findings related to assessment in general chemistry lectures both theory and laboratory further emphasizes the need for a thorough and systematic literature review. This research aims to address the gap by conducting a systematic literature review on assessment strategies applied in general chemistry lectures and laboratory. In more detail, this study aims to: (1) identify the assessment strategies used, (2) analyze the effectiveness of these strategies in improving student learning outcomes, and (3) provide insights that can support educators and policy makers in designing more innovative and effective assessment methods in general chemistry lectures and laboratory. The findings of this review are expected to enrich the literature by providing evidence-based guidance that supports improved student engagement and learning outcomes in chemistry education. To achieve the objectives of this study, the researcher formulated the following research questions:

(a) RQ1. What are the research trends in assessment practices in general chemistry learning and laboratory education during 2020–2024?
(b) RQ2. What assessment strategies and instruments have been developed in general chemistry learning and laboratory contexts?
(c) RQ3. What are the emerging trends and key challenges in general chemistry assessment practices?

## 2. Methodology

This study employs a Systematic Literature Review (SLR) methodology, following the Preferred Reporting Items for Systematic Reviews and Meta-Analyses (PRISMA) 2020 guidelines ( Page et al., 2021). The SLR method is a structured and replicable process of identifying, evaluating, and synthesizing all relevant research evidence pertaining to a specific topic ( Hu et al., 2022; Xiao & Watson, 2019). This approach was selected because it enables transparent and systematic identification of assessment practices in general chemistry education, minimizes selection bias, and provides a comprehensive synthesis of existing evidence. The PRISMA framework offers clear procedural steps identification, screening, eligibility assessment, and inclusion that ensure both the scientific rigor and replicability of the review process ( Kitchenham, 2004; Page et al., 2021). The protocol for this systematic literature review was registered retrospectively on the Open Science Framework (OSF) with the DOI: 10.17605/OSF.IO/HSMEA. The overall review process is illustrated in the PRISMA flowchart presented in Figure 1.

**Figure 1.**
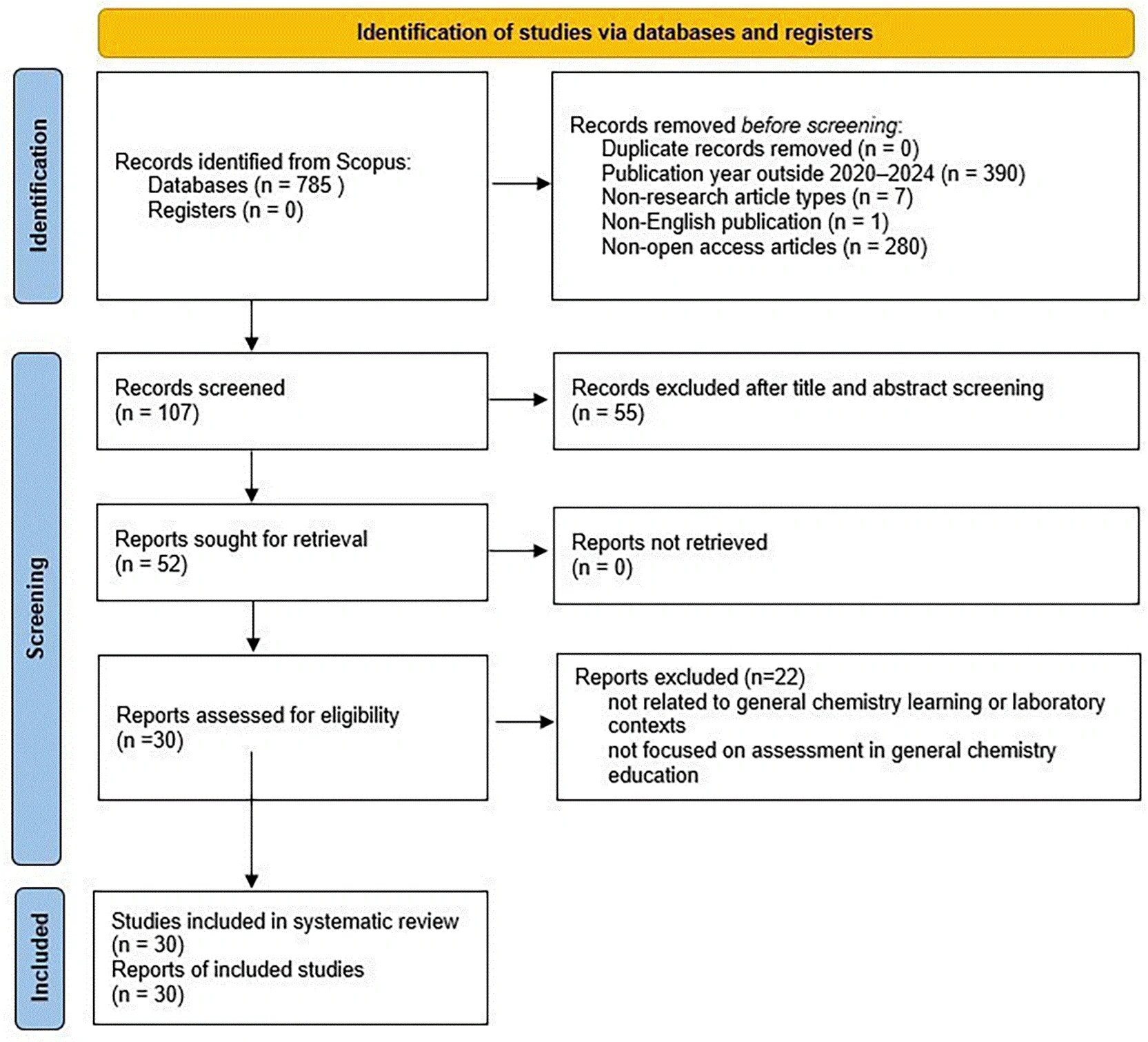
PRISMA 2020 flow diagram of the study selection process.

### 2.1 Review design

This study is designed as a qualitative systematic literature review using a narrative synthesis approach. The review was conducted to explore, map, and synthesize research findings related to assessment strategies in general chemistry theory and laboratory courses published between 2020 and 2024. A narrative synthesis was chosen over a quantitative meta-analysis given the heterogeneity of the included studies in terms of research design, assessment instruments, and outcome measures, which makes statistical pooling inappropriate ( Xiao & Watson, 2019). The review was guided by three main research questions: (1) What are the research trends in assessment practices in general chemistry learning and laboratory education during 2020–2024? (2) What assessment strategies and instruments have been developed in general chemistry learning and laboratory contexts? (3) What are the emerging trends and key challenges in general chemistry assessment practices? These questions were formulated at the outset of the review to provide a clear framework for article searching, screening, and data extraction.

### 2.2 Search strategy

A systematic search for relevant articles was conducted using the Scopus database, one of the most comprehensive multidisciplinary academic databases widely used in education research ( Cronin et al., 2008). The search was executed on November 3, 2024. Scopus was selected as the sole database for this review due to its broad coverage of high-quality, peer-reviewed journals in the field of chemistry education and its robust filtering capabilities. The search query was constructed using Boolean operators (AND, OR) to account for the diversity of terminology used across the literature. Key concepts were identified and expanded to include synonymous terms: assessment was broadened to include evaluation, grading, and feedback; general chemistry was extended to introductory chemistry and basic chemistry; and laboratory was paired with practical and experiment, while lecture was also represented by classroom and course. The final search query, executed in the TITLE-ABS-KEY fields of Scopus, was as follows:

*TITLE-ABS-KEY((“assessment” OR “evaluation” OR “grading” OR “feedback”) AND (“general chemistry” OR “introductory chemistry” OR “basic chemistry”) AND (“lecture” OR “classroom” OR “course” OR “laboratory” OR “practical” OR “experiment”))*

This search query yielded a total of 785 articles. These results were then subjected to a structured screening process based on the eligibility criteria described in Section 2.3.

### 2.3 Eligibility criteria

Eligibility criteria were established prior to the search to ensure that only studies relevant to the focus of this review were included, thereby minimizing bias in the selection process ( Lin et al., 2018). The criteria were categorized into inclusion and exclusion criteria as detailed in Table 1.

**Table 1.** Inclusion and exclusion criteria for article selection.

Inclusion criteria	Exclusion criteria
Published between 2020 and 2024	Scope outside general chemistry learning and laboratory (e.g., high school, graduate-level, other science disciplines)
Scope of research on general chemistry learning and/or laboratory at the undergraduate/college level	Incomplete article structure (e.g., missing results or methods section)
Accessible in open access format	Publication type other than original research articles (e.g., review articles, book chapters, editorials, conference proceedings)
Written in English	Written in a language other than English
Original empirical research articles (quantitative, qualitative, or mixed methods)	

These criteria were applied consistently across all stages of the article screening process to ensure methodological transparency and reproducibility.

### 2.4 Study selection process

The study selection process was conducted in four sequential stages following the PRISMA 2020 framework: identification, screening, eligibility assessment, and inclusion ( Page et al., 2021). To ensure objectivity and minimize subjectivity in article selection, all screening and eligibility assessment stages were carried out independently by three reviewers. Inter-rater agreement was calculated as the percentage of cases in which all three reviewers reached identical decisions, as well as Fleiss’ Kappa (κ), which is the appropriate statistic for assessing agreement among three or more raters. A 100% agreement rate (κ = 1.00) was achieved at both the title/abstract screening stage and the full-text eligibility assessment stage, indicating that all three reviewers applied the eligibility criteria consistently and without interpretive discrepancy. This unanimity reflects the operational clarity and specificity of the inclusion and exclusion criteria established prior to screening (Section 2.3), which left minimal room for subjective judgment. As complete agreement was reached across all three reviewers at every stage, no further consensus procedure or arbitration was required.

**Identification.** A total of 785 articles were identified through the Scopus database search. No additional records were retrieved from other sources or databases.

**Screening.** All 785 records were initially screened based on their titles and abstracts. Articles that were clearly irrelevant to the topic of assessment in general chemistry or did not meet the basic eligibility criteria (e.g., published before 2020, written in languages other than English, or categorized as non-research publications) were excluded at this stage. All three reviewers reached identical decisions for all 785 records, yielding a percentage agreement of 100% (Fleiss’ κ = 1.00).

**Eligibility assessment.** The full texts of potentially eligible articles were retrieved and assessed in detail against all inclusion and exclusion criteria outlined in Section 2.3. Articles whose scope extended beyond general chemistry assessment, had incomplete structures, or were categorized as review articles or book chapters were excluded at this stage, with reasons documented for each excluded article. Complete agreement was again reached among all three reviewers across all full-text eligibility decisions, yielding a percentage agreement of 100% (Fleiss’ κ = 1.00).

**Inclusion.** Following the full-text assessment, a final set of 30 articles met all eligibility criteria and were included in the review. These articles were subsequently subjected to systematic data extraction and analysis. The complete article selection process is illustrated in the PRISMA flowchart in Figure 1.

### 2.5 Quality appraisal

Quality appraisal was not formally conducted in this review. This decision was made on the basis of several methodological considerations. First, the primary aim of this review is descriptive and exploratory to map and synthesize existing research trends, assessment strategies, and innovations in general chemistry education rather than to evaluate the effectiveness of a specific intervention or treatment. In such descriptive systematic reviews, formal risk of bias assessment is generally considered less applicable, as the focus lies on characterizing the literature rather than estimating effect sizes or causal relationships ( Xiao & Watson, 2019). Second, all 30 included articles were sourced exclusively from Scopus-indexed, peer-reviewed journals, the majority of which carry Q1 or Q2 quartile rankings including the Journal of Chemical Education and Chemistry Education Research and Practice. Indexing in Scopus itself serves as a baseline indicator of scholarly quality, as it requires journals to meet rigorous editorial and peer-review standards ( Kitchenham, 2004). Third, the heterogeneity of research designs across the included studies spanning instrument development, quasi-experimental, descriptive, and qualitative approaches makes the application of a single standardized appraisal tool such as CASP or the Mixed Methods Appraisal Tool (MMAT) methodologically inappropriate without significant adaptation. The absence of formal quality appraisal is acknowledged as a limitation of this review and is discussed further in limitation studies section.

### 2.6 Data extraction

Data extraction from the 30 included articles was conducted systematically using a structured extraction framework. For each article, the following information was extracted: (1) article code, author(s), and year of publication; (2) article title; (3) country of origin; (4) journal source and quartile ranking; (5) research focus (learning assessment, laboratory assessment, or integration of both); (6) purpose of assessment (formative, summative, or both); (7) assessment components measured (e.g., conceptual understanding, scientific argumentation, metacognition); (8) assessment instruments used (e.g., multiple-choice questions, rubrics, concept maps, inventories, questionnaires); (9) assessment methods employed (e.g., written tests, electronic-based assignments, peer assessment, practical tests); and (10) key research findings. The extracted data were organized into a structured summary table ( Table 2 in the Results section) to facilitate cross-study comparison and thematic synthesis. To ensure the credibility and accuracy of extracted data, all articles underwent an in-depth reading and critical appraisal process. Any ambiguities regarding categorization were resolved through discussion among the research team, and data were verified by cross-referencing with the original articles.

### 2.7 Data analysis

Data analysis in this study was conducted using narrative synthesis, which is appropriate given the heterogeneous nature of the included studies in terms of design, context, and outcome measures ( Xiao & Watson, 2019). The narrative synthesis involved thematic grouping and interpretive analysis of the extracted data to identify patterns, trends, and gaps across the reviewed literature. The analysis was organized around the three research questions of this review. **For RQ1**, articles were analyzed and grouped based on: (a) country of origin, (b) journal source and quartile ranking, (c) year of publication, (d) focus of assessment (learning, laboratory, or integrated), and (e) purpose of assessment (formative, summative, or combined). Descriptive statistics in the form of frequencies and distributions were used to summarize these characteristics and are presented through figures and tables. **For RQ2**, the extracted information on assessment components, instruments, and methods was thematically coded and organized into a classification matrix ( Table 3). This process allowed for the identification of commonly used and emerging assessment strategies across the reviewed studies. **For RQ3**, the analysis focused on identifying overarching trends in assessment innovation particularly the role of digital technology, as well as persistent challenges and future directions in general chemistry assessment. Cross-study patterns were identified through iterative reading and thematic grouping of the findings across all 30 articles. Throughout the analysis process, the credibility of the synthesis was maintained through meticulous documentation, consistent application of the analytical framework, and cross-verification of key interpretive claims with the source articles ( Kitchenham, 2004).

## 3. Results and Discussion

### 3.1 RQ1: What are the research trends in assessment practices in general chemistry learning and laboratory education during 2020–2024?

Research on assessment in general chemistry theory and laboratory courses has become one of the main focuses in improving the quality of chemistry learning. Various assessment strategies and instruments continue to be developed to address learning needs, including in improving students’ understanding of concepts, laboratory skills, and their active involvement in the learning process. These new approaches often utilize technology, diverse assessment formats and metacognitive components to ensure better learning outcomes.

**Figure 2.**
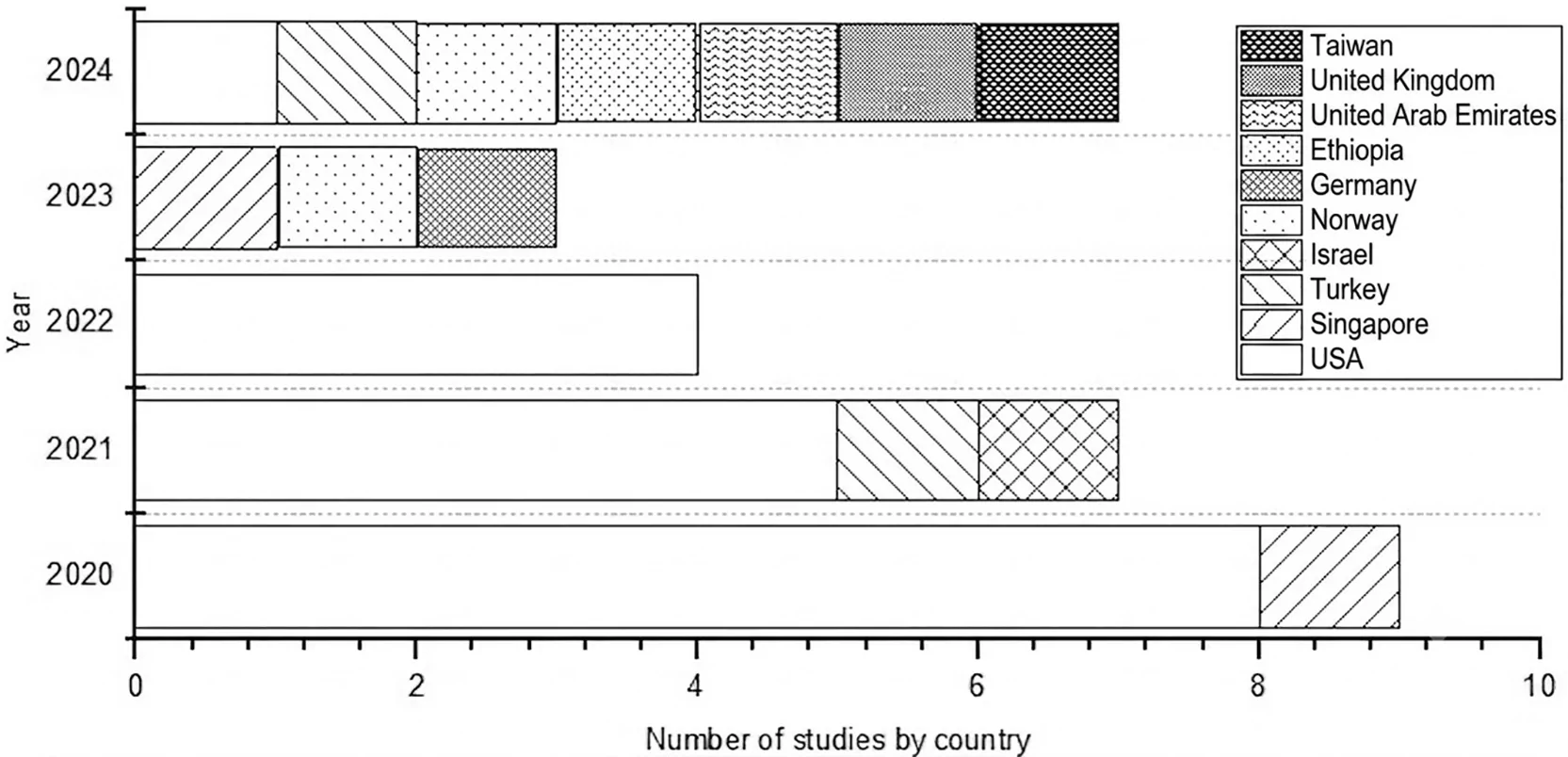
Number of studies by Countries (2020–2024).

A summary of recent research results on assessment in general chemistry theory and laboratory courses published in the Scopus database over the past five years is provided in Table 2. These studies cover the development of innovative assessment tools, the application of technology in the assessment process, as well as its impact on practical skills, conceptual understanding, and student learning motivation. The results of this search provide insights into how assessment methods can support chemistry learning more effectively and be relevant to current educational needs.

**Table 2.** Summary of assessment research results in general chemistry theory and laboratory lectures published in the Scopus database for the last 5 years (2020–2024).

Article code	Author (Year)	Article title	Research findings
A1	Ye et al. (2020)	The Impact of Coupling Assessments on Conceptual Understanding and Connection-Making in Chemical Equilibrium	The combination of concept maps and creative exercises helps improve students’ ability to explain and connect chemical concepts.
A2	Sweeder & Herrington (2020)	Formative Assessments Using Text Messages to Develop Students’ Ability to Provide Causal Reasoning in General Chemistry	The use of e-text messaging assignments improves students’ causal reasoning and success on final exams.
A3	Talbert et al. (2020)	Revisiting the Use of Concept Maps in a Large Enrollment General Chemistry Course	The concept map assessment helped students to demonstrate better concept linking skills although the difference in results was not statistically significant.
A4	Koevoets-Beach et al. (2023)	“I guess it was more than just my general knowledge of chemistry”: exploring students’ confidence judgments in two-tiered assessments	Assessment with two-level test questions can explore students’ self-confidence. However, a level of confidence that is not solely dependent on content understanding may ultimately inhibit students’ metacognition.
A5	Atkinson et al. (2020)	Development of the Reaction Coordinate Diagram Inventory	This assessment can be used to test students’ understanding and misconceptions, differentiate students according to their level of understanding, identify student difficulties, identify concepts that are difficult to understand.
A6	Aguirre-Mendez et al. (2020)	Predicting Components of Argumentative Writing and Achievement	Assessment techniques and instruments can identify students’ scientific argumentation skills that can improve students’ understanding of chemical concepts and learning outcomes.
A7	Stephenson et al. (2020)	Development and Validation of Scientific Practices Assessment Tasks for the General Chemistry Laboratory	The assessment instrument has high validity to measure students’ scientific skills in general chemistry laboratory activities.
A8	Lau (2020)	Enhancing Formative and Self-Assessment with Video Playback to Improve Critique Skills in a Titration Laboratory	The use of this assessment showed an increase in students’ understanding and ability to assess titration skills in others compared to participants who did not use the checklist. This assessment technique also received positive responses from students.
A9	Hensen et al. (2020)	Assessing Differences between Three Virtual General Chemistry Experiments and Similar Hands-On Experiments	The assessment results show differences in student achievement in several learning objectives. However, some students still experience problems in participating in assessments that involve technology due to their lack of knowledge in operating the application.
A10	Edwards (2021)	Out-of-Class Assessment Activities for Feedback in a General, Organic, and Biochemistry Course	The “Daily Feedback” task allows students to identify concepts that have been mastered so that students can focus on concepts that have not been mastered. The use of this instrument also received a positive response from students.
A11	Balabanoff et al. (2021)	Development of the Water Instrument: A Comprehensive Measure of Students’ Knowledge of Fundamental Concepts in General Chemistry	This valid and reliable “Water Instrument” can be used to measure students’ conceptual understanding of general chemistry concepts.
A12	Kapici et al. (2022)	Comparison of the Quality of Written Scientific Arguments in Different Laboratory Environments	This assessment method and instrument proved to be able to improve students’ ability to write their scientific argumentation both in direct laboratory and virtual laboratory.
A13	Yik et al. (2021)	Development of a Machine Learning-Based Tool to Evaluate Lewis Acid–Base Model Use	The use of these technology-assisted formative assessments allows students to reflect on their achievements in summative assessments and makes assessment faster and more efficient.
A14	Zemel et al. (2021)	Preservice Teachers’ Enactment of Formative Assessment Using Rubrics	The application of formative assessment in inquiry-based laboratories has great potential to support students’ conceptual learning and critical thinking skills, especially in analyzing experimental data.
A15	Hosbein et al. (2021)	Development of the Investigation Design, Explanation, and Argument Assessment for General Chemistry I Laboratory	The instrument developed has strong validity and reliability to measure students’ understanding of the practices carried out in the laboratory, especially in the aspects of investigation design, explanation, and scientific argumentation.
A16	Zhang & Wink (2021)	Examining an Acid−Base Laboratory Practical Assessment from the Perspective of Evidence-Centered Design	The evidence-based assessment provided in-depth insight into students’ multidimensional skill patterns in the acid- base titration laboratory and revealed that most students only demonstrated understanding at the process rather than conceptual level and that there were limitations in connecting the use of data to a broader chemical context.
A17	Muteti et al. (2022)	Blending Muddiest Point Activities with Common Formative Assessments	Muddiest point activities with formative assessments have been shown to improve the performance of minority and first-generation students compared to other students.
A18	Shah et al. (2022)	Investigating the Impact of Assessment Practices on Students Perceived to Be at Risk	Tests using multiple-choice questions still require mathematical calculation skills and prove difficult for students, so diversification of question types is needed to help reduce the gap in student performance.
A19	Fringer et al. (2022)	Steps in the Right Direction: A Quasi-experimental Comparison of an Inquiry-Based and a Traditional Laboratory	This assessment proved to be able to identify students’ abilities and perceptions in designing experiments and students’ skills in carrying out inquiry-based and conventional experiments.
A20	Hosbein & Walker (2022)	Assessment of Scientific Practice Proficiency and Content Understanding Following an Inquiry-Based Laboratory Course	The IDEAA-GC1 assessment instrument is proven to be a valid instrument to evaluate students’ science process skills in general chemistry laboratory, especially in the aspects of designing experiments and composing scientific arguments. However, limited understanding of chemical concepts is still one of the obstacles to improving students’ science process skills.
A21	Beerepoot (2023)	Formative and Summative Automated Assessment with Multiple-Choice Question Banks	The question bank can be used for formative assessments, supporting active student learning in large online classes, out-of-class assessments, and summative assessments through online exams. Automated assessments enable quick feedback that enhances students’ learning independence and supports continuous mastery of concepts.
A22	Eitemüller et al. (2023)	Digitalization of Multistep Chemistry Exercises with Automated Formative Feedback	The use of this digital formative test received a positive response from students as they were given the opportunity to correct their answers even in multiple attempts. Automatic feedback on the tiered task improved student performance compared to the manual method.
A23	Lau et al. (2022)	Integrating Chemistry Laboratory–Tutorial Timetabling with Instructional Design and Its Impact on Learner Perceptions and Outcomes	This assessment method is able to identify the improvement of students’ titration skills and concept understanding. The instrument used can also reveal the cognitive load possessed by students.
A24	Hagos & Andargie (2024)	Effects of Formative Assessment with Technology on Students’ Meaningful Learning in Chemistry Equilibrium	The involvement of technology in formative assessment is proven to improve conceptual and procedural chemistry understanding and student engagement compared to students using conventional formative assessment.
A25	Ling et al. (2024)	Computer Quiz Games in General Chemistry for Engineering Majors in an ESL Environment	The application of quizzes in learning both computer- assisted and written helped improve learning outcomes compared to classes that did not use quizzes. in addition, there was an increase in students’ positive attitudes, they were more motivated and enjoyed lectures and helped them learn new concepts in chemistry.
A26	Shepherd & Garrett-Roe (2024)	Low-Stakes, Growth-Oriented Testing in Large-Enrollment General Chemistry 1	The assessment method developed is effective in helping students develop and learn in large classes, students who may be less successful at the beginning still have the opportunity to improve. Results show an increase in student motivation and a gradual improvement in learning outcomes through retake assessments.
A27	Huynh & Yang (2023)	Impact of Fill-in-the-Nodes Concept Maps on Low Prior-Knowledge Students’ Learning	The use of concept maps is proven to improve student learning outcomes. This method also received a positive response from students because in addition to being an assessment tool, concept maps help them understand concepts.
A28	Kaya & Kaya (2024)	The Impact of Co-Design-Based Formative Assessment Practices on Preservice Science Teachers’ Understanding	Collaborative assessment design using concept map resulted in a significant increase in concept understanding. This assessment also proved to be able to reduce misconceptions related to general chemistry concepts in the laboratory.
A29	Østby et al. (2024)	Authentic Hands-On Learning Approaches for Bulky Student Cohorts in General Chemistry Laboratory	This research shows that this assessment method provides a relevant understanding between chemistry and life contexts. Students also considered that the assessment criteria provided were very helpful in the process of writing and evaluating reports.
A30	Coyte & Lowry (2024)	Paired Online Laboratory Assessments: Formative Engagement and Summative Attainment	Students who participated in formative assessments showed higher achievement in summative than those who did not participate in formative assessments.

**Table 3.** Assessment strategies in chemistry learning and laboratory.

Assessment components	Assessment instrument	Assessment method
Understanding and connecting concepts	1. Concept map (A1, A3, A27, A28) 2. Written questions: description (A16), multiple choice (A3, A27) and graded diagnostic (A6, A11, A18 3. Electronic based questions: descriptions (A2, A13, A16, A17, A26), multiple choice (A17, A21, A22, A24, A25, A30), Inventory (MLII) (A5, A23) 4. Assessment rubrics: scientific argumentation (A6, A7, 12), laboratory reports (A9, A16, A29) daily notes (A10) and worksheets (A30)	1. Written assignments: concept map creative exercises (A1), lab reports (A9, A16, A29), experiment-based assignments (A7), 2. Electronic-based assignments: electronic text message-based assignments (A2), daily notes (A10), concept maps (A1, A27, A28) 3. Written testing (A3, A6, A7, A11, A12, A18, A23) 4. Electronic-based testing (A5, A10, A13, A21, A22, A24, A25, A26, A27, A30) 5. “Muddiest point” activity with formative test (A17)
Procedural and conceptual understanding, Causal reasoning	1. Electronic-based questions: Open- ended essay (A2), and multiple choice (A22, 24). 2. observation guidelines (A24) 3. interview guidelines (A24)	1. Electronic text message-based assignment (A2), 2. observation (A24) 3. interview (A24)
Metacognition, misconceptions and learning difficulties	1. Written questions: multiple choice and descriptive (A17, graded diagnostic (A4) 2. Interview question (A4) 3. Assessment rubric: electronic diary (A10), blueprint practice exams sheet (A10), concept map (A28).	1. Electronic-based assignments: electronic diary (A10), blueprint practice exams sheet (A10), concept map (A28). 2. Written testing (A17, A4) 3. interview (A4) 4. “Muddiest point” activity with formative test (A17)
Scientific argumentation	1. Written questions: laboratory-based descriptions (A7, A12), IDEAA- GC1 in the form of graded short description questions (A15, A20) 2. IDEAA-GC1 assessment rubric (A15, A20)	1. Assignment: laboratory assignment with evidence-centered design approach (A7) 2. Written testing (A12, A15, A20) 3. Practical lab, (A7)
Scientific practice and experimental data analysis	1. Written Questions: open-ended description based on laboratory (A7), description based on evidence-centered design (A16) 2. Rubric: laboratory report (A14, A16, A29) 3. student reflection notes (A29)	1. Assignment: laboratory assignment with evidence-centered design approach (A7), lab report (A14, A16, A29) 2. Peer assessment (A29) 3. Laboratory practice (A7)
Design and conduct experiments	1. Written questions: lab-based descriptions (A7, A8, A20), IDEAA-GC1 (A15, A20), 2. experimental design skills test (EDAT)(A19) 3. Electronic based questions: Inventory (MLII) (A23), multiple choice (A30) 4. Rubrics: IDEAA-GC1 (A15, A20), 5. laboratory video analysis sheet (A8), laboratory report (A30) 6. Observation checklist (A23) 7. Worksheet (A30)	1. Written assignments: experiment- based assignments (A7), laboratory video analysis (A8), laboratory report (A29), worksheet (A30) 2. Written testing (A15, A19, A20) 3. Electronic-based testing (A23, A30) 4. Peer assessment and self- assessment (A23, A29) 5. Practical test (A7) 6. MLII survey (A23)
Affective experience in the laboratory	1. Affective questionnaire (A9, A19) 2. Student self-confidence interview question (A4) 3. Self-assessment checklist (A8)	1. Questionnaire (A9, A19) 2. Self-assessment (A8) 3. interview (A4)
Self-regulation and motivation skills	Questionnaire (A21, A25)	Questionnaire (A21, A25)

Based on the results of the analysis and extraction of articles related to the assessment of general chemistry lectures and laboratories, some information was obtained, among others:

*Article Distribution by Author Country*

Based on Figure 2 above, it can be seen that research related to the assessment of general chemistry lectures and laboratory in the last 5 years is still dominated by developed countries such as the United States with a total of 18 publications. Then followed by other developed countries with two articles each, namely Norway and Singapore ( Lau, 2020; Lau et al., 2022), Jerman, Inggris, Taiwan, Uni Emirat Arab. Germany, UK, Taiwan, United Arab Emirates. Looking at the number of publications in the last 5 years, it can be seen that although the number of publications decreased from 2020 (n = 9) to 2021 (n = 7), 2022 (n = 4), 2023 (n = 3), the number of publications increased again in 2024 (n = 7). This shows a growing interest in developing general chemistry assessments in various parts of the world. It can be seen that in 2024 there was an increase in the number of publications and contributions from various countries in publishing articles related to general chemical assessments.

Based result of analysis, it can be seen that research related to the assessment of general chemistry lectures and laboratory in the last 5 years is still dominated by developed countries such as the United States with a total of 18 publications. Then followed by other developed countries Norway and Singapore, Jerman, Inggris, Taiwan, Uni Emirat Arab. Germany, UK, Taiwan, United Arab Emirates. It is undeniable that these developed countries have advantages in educational infrastructure and access to adequate technology that allows for the development of innovative assessment instruments. Apart from dominating in terms of the number of articles, the United States also dominates in terms of innovative assessment development involving technology such as digital question banks and machine learning-assisted tests. Developing countries such as Ethiopia and Turkey tend to focus on adapting innovative assessment methods that are more affordable and relevant to the local context.

Based on the number of publications in the last 5 years, it can be seen that although the number of publications decreased from 2020 to 2024. This shows a growing interest in developing general chemistry assessments in various parts of the world. It can be seen that in 2024 there was an increase in the number of publications and contributions from various countries in publishing articles related to general chemical assessments. However, there is still a gap between developed and developing countries regarding access and use of technology in chemistry learning assessment. Therefore, international collaboration is needed to expand access and research capacity in other regions and ensure inclusivity in the development of assessment methods.

*Article Distribution by Journal source in the last 5 years*

Based on the Figure 3, it can be seen that articles related to this theme are still concentrated in highly reputable journals such as Journal of Chemical Education, Q2 (n = 15) and Chemistry Education Research and Practice, Q1 (n = 12) then followed by International Journal of Science and Mathematics Education, Q1 (n = 1), Journal of Science Education and Technology, Q1 (n = 1) and Canadian Journal of Chemistry, Q3 (n = 1). This indicates that this article is a quality article because of the journal’s reputation that relies on editorial rigor and other scientific contributions so that it can be said that this literature review has high credibility and relevance to global needs in the field of education and gets attention from the academic community (lecturers, researchers, educational practitioners).

**Figure 3.**
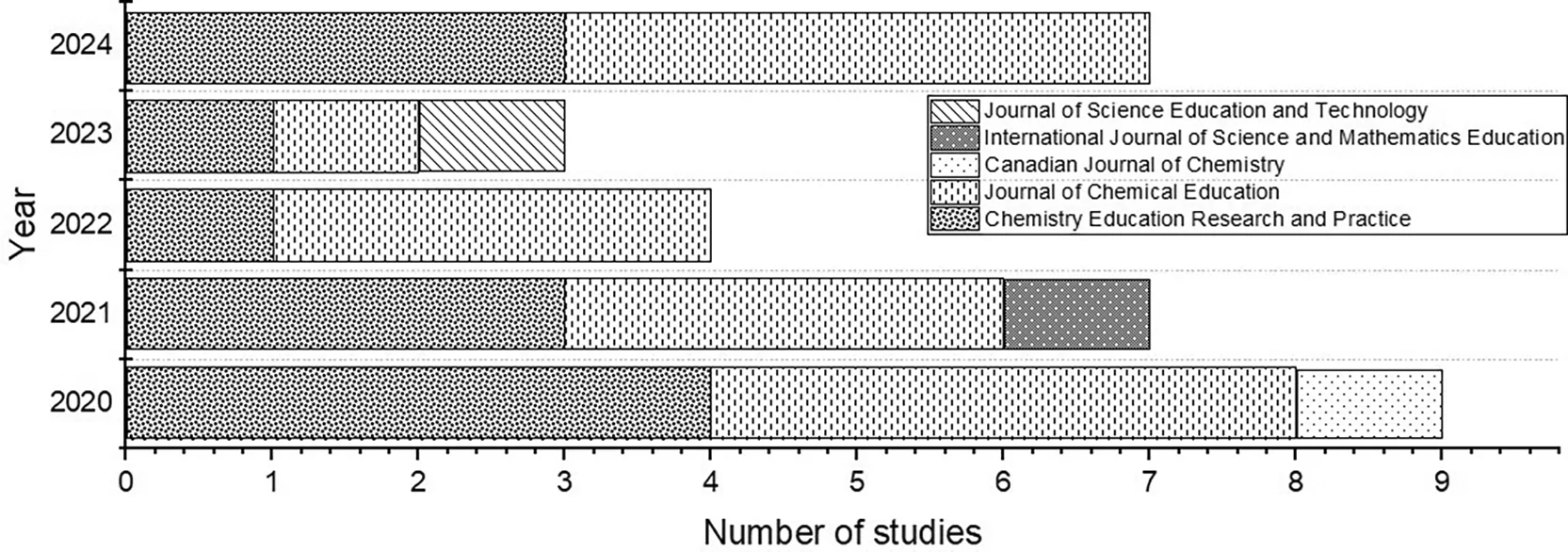
Number of Studies by Journal Publisher (2020–2024).

Based on article distribution by journal source in the last 5 years, it can be seen that articles related to this theme are still concentrated in highly reputable journals such as Journal of Chemical Education, Chemistry Education Research and Practice, International Journal of Science and Mathematics Education, Journal of Science Education and Technology and Canadian Journal of Chemistry. This indicates that this article is a quality article because of the journal’s reputation that relies on editorial rigor and other scientific contributions so that it can be said that this literature review has high credibility and relevance to global needs in the field of education and gets attention from the academic community (lecturers, researchers, educational practitioners). These highly reputable journals are one of the platforms used to disseminate innovations in assessment methods, ranging from conventional assessment with various approaches to assessment digitization. In addition, the fact that most of the articles come from journals with high reputation and quality shows the importance of validity and impact of research on chemistry learning.

*Article distribution based on aspects of assessment conducted (learning or laboratory only or learning and laboratory)*

The distribution of articles related to learning assessment and general chemistry laboratory shown in Figure 4 reflects the focus of research in the last 5 years. Based on the focus of the assessment carried out, the *authors* are grouped into: learning assessment, laboratory assessment, and a combination of learning and laboratory assessment. In the last five years, articles focusing on learning assessment (n = 17) still dominate compared to practical assessment (n = 13).

**Figure 4.**
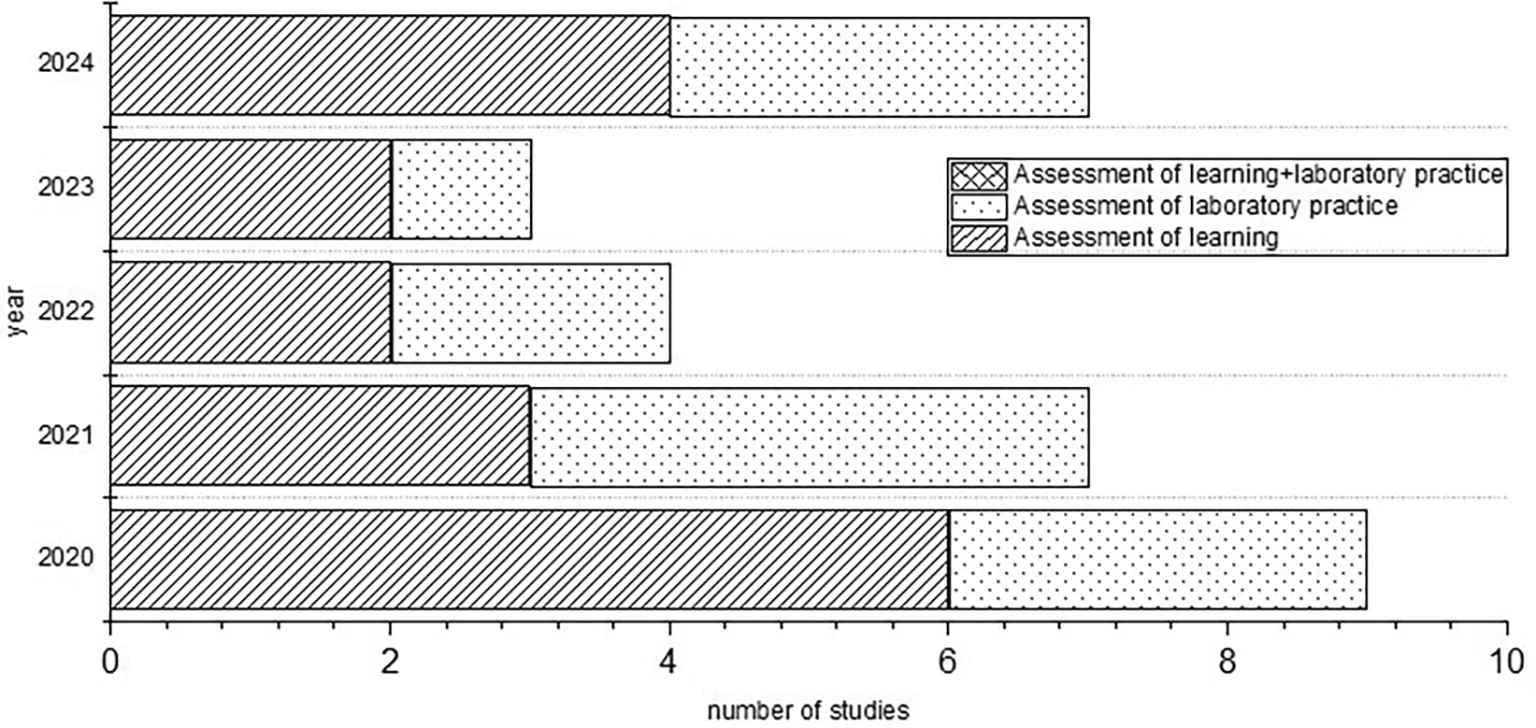
Number of Studies by the focus of the assessment conducted.

In the last 5 years, the distribution of articles related to learning assessment and general chemistry laboratory based on the focus of the assessment carried out articles focusing on learning assessment still dominate compared to practical assessment. Chemistry, which is one of the branches of science, is characterized by an inquiry process that can be carried out through laboratory. Laboratory is an inseparable part of chemistry learning, laboratory is an important and essential part of chemistry learning that helps students improve mastery of concepts ( McKnelly et al., 2020; Watts et al., 2022) and train various skills ( Keiner & Graulich, 2021) ( Greenstein, 2012; Lieber et al., 2022), and gain hands-on experience ( Petritis et al., 2021; Singh et al., 2021). Through laboratory students are given the opportunity to make direct observations, explore concepts, develop skills and understand the object of study ( Young, 2021; M. (John) Zhang et al., 2020). Therefore, the assessment carried out not only focuses on theoretical learning but also practical assessment as part of the inquiry process and integrates learning and laboratory. However, research integrating learning assessment and general chemistry laboratory in the last 5 years is still very limited, especially in highly reputable international journals. This condition opens opportunities for other researchers to make the integration of learning and laboratory assessment in general chemistry lectures as the main focus, considering that both are two complementary components in supporting holistic and effective general chemistry learning ( Nahadi et al., 2023; Penn & Mavuru, 2020).

*Article distribution based on the purpose of the assessment (formative or summative)*

Scriven (1966) was the one who introduced the terms formative and summative assessment to distinguish assessment *based on* purpose. Formative assessment is used to improve the learning process (assessment for learning) while summative assessment aims to provide decisions and summaries of student achievement (assessment of learning) ( Alexander et al., 2020). Figure 5 shows the distribution of articles based on the purpose of assessments conducted in the last 5 years. Formative assessment still dominates in the last 5 years, indicating that formative assessment is considered as one of the main approaches that support general chemistry learning and laboratory.

**Figure 5.**
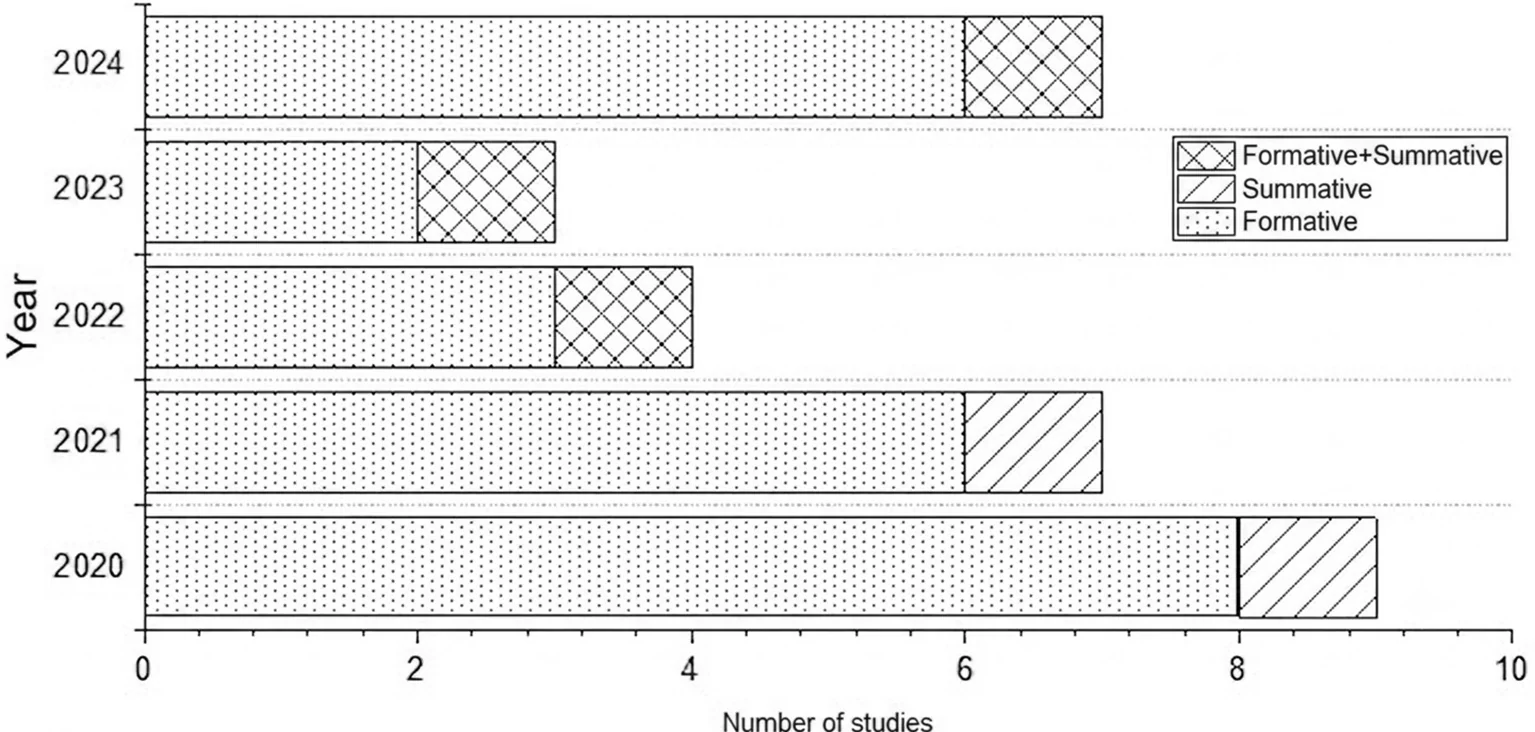
Number of studies by the purposed of the assessment conducted.

Based on the purpose of assessments conducted in the last 5 years, formative assessment still dominates in the last 5 years, indicating that formative assessment is considered as one of the main approaches that support general chemistry learning and laboratory. Formative assessment is an assessment process carried out during the learning process with the main purpose of providing feedback to students and teachers in order to improve the learning process and increase student understanding of the material being studied ( Abell & Sevian, 2021; Nahadi, N. et al., 2015). Formative assessment is considered effective because it can provide immediate feedback and help students improve their understanding ( Alotaibi, 2019; Shepard et al., 2018; Zemel et al., 2021).

On the other hand, summative assessment is a form of assessment that aims to determine the extent to which students can achieve learning objectives so as to obtain final decisions regarding learning outcomes such as graduation, grade promotion and progress reports ( Cobbold & Wright, 2021; Mahyan et al., 2020). This assessment is often used for administrative purposes and is more formal in nature and is generally implemented in the form of tests, final projects, or portfolios, with a focus on documenting the final results rather than the learning process ( Burrows et al., 2021; Veale et al., 2020). Based on the results of the analysis, the number of articles that discuss summative assessment and the combination of formative and summative is still limited. Whereas summative assessment also plays an important role in measuring student learning outcomes. so does the combination of formative and summative assessments. Therefore, more attention is needed from researchers to develop more holistic and balanced assessments, by integrating formative and summative aspects effectively, especially in the context of general chemistry learning and laboratory. This approach is important to ensure that assessments not only assist the learning process but also provide a comprehensive evaluation of student learning outcomes.

### 3.2 RQ2: What assessment strategies and instruments have been developed in general chemistry learning and laboratory contexts?

By utilizing various instruments and methods, the assessment approach in chemistry learning focuses not only on the end result, but also on the student learning process. This research makes an important contribution to the chemistry education literature by identifying various assessment instruments and methods that can support student learning and skill development. Chemistry learning requires an assessment approach that can measure various aspects of students’ cognitive, affective and psychomotor learning outcomes. Assessment in chemistry learning both theoretical and practical learning plays an important role in evaluating conceptual and procedural understanding, causal reasoning, metacognition, misconceptions, and learning difficulties, scientific argumentation, scientific practice and experimental data analysis, designing and conducting experiments, affective experiences in the laboratory, as well as self-regulation and motivation skills ( Alkan, 2013; Sutiani et al., 2021). Each of these components is measured through a combination of instruments and assessment methods designed to ensure the assessment provides valid and reliable data and is able to identify student learning needs and outcomes. This assessment is supported by instruments such as multiple choice questions, descriptions, diagnostics, concept maps, questionnaires, checklists, inventories, assessment rubrics to assess diaries, concept maps, laboratory reports, worksheets.

In detail, information related to assessment components, instruments and assessment methods that can be used in general chemistry learning and laboratory in the last 5 years is in Table 3.

Based on the results of the article analysis, assessment is mostly dominated by the use of instruments such as multiple choice questions and description questions and assessment methods in the form of tests or written or electronic-based tasks. Multiple choice questions and descriptions can be used to evaluate students’ ability to explain and apply concepts in depth. Although many opinions state that multiple choice questions can only be used to measure low-level thinking skills, in several studies it has been proven that multiple choice questions can be used to measure higher- level thinking skills by combining them with limited description and open description questions so that they become multilevel choice questions and are proven to be able to measure students’ concept understanding at the C1-C6 level ( Bachtiar et al., 2020; Chandrasegaran A. L et al., 2007; Keller & Hermanns, 2024). Multiple choice questions and descriptions are proven to not only be able to reveal concept understanding but also causal reasoning, metacognition, misconceptions, and learning difficulties, scientific argumentation, analyzing experimental data and designing experiments ( Koevoets-Beach et al., 2023; Muteti et al., 2022; Sweeder & Herrington, 2020). Concept maps are also a form of instrument that is not only used to measure students’ concept understanding, concept maps can also provide an overview of how students organize their knowledge and connect various concepts that have been learned and express their scientific arguments in graphical form ( Kaya & Kaya, 2024; Bruce et al., 2023; Vachliotis et al., 2021). Another form of instrument that is still rarely used in measuring concept understanding is electronic-based inventories. This inventory can also be used to assess students’ ability to plan and conduct experiments ( Atkinson & Bretz, 2021; Lau, 2020). An inventory is a series of questions or statements designed to collect information from students both quantitatively and qualitatively such as concept understanding, misconceptions, metacognition, certain skills ( Jack, 2013; Marks et al., 2022). As a form of assessment instrument, inventory has advantages such as its efficient and flexible use because it can be used to collect data in a short time and provide valid and reliable data ( Bretz S. & Murata Mayo A., 2018; McAlpin et al., 2023; Rana & Mahmood, 2010).

In laboratory assessment, rubrics are widely used to assess daily notes, laboratory reports, worksheets and concept maps. This rubric is used as a reference in assessing so that the assessment is more objective, measurable and makes it easier to give scores or final results ( Aydin-Gunbatar & Akin, 2022; Bylander & Gustafsson, 2021; Hadas et al., 2023; Yik et al., 2022). In several research articles the assessment rubric is used as a reference for assessing scientific argumentation, laboratory final reports, daily notes ( Edwards, 2021) and student worksheets ( Coyte & Lowry, 2024) both in writing and electronically based. The results of the assessment using this rubric can not only assess students’ conceptual and procedural understanding but can also be used to assess students’ ability to reflect on their learning process and integrate theoretical knowledge with experimental procedures ( Flesch et al., 2024; Nainggolan et al., 2020), identify student learning difficulties ( Asmussen et al., 2023; Stroumpouli & Tsaparlis, 2022) and plan strategies for improving students’ understanding and abilities. This is in line with previous relevant research related to the use of rubrics as an assessment instrument for chemistry learning ( Schmid et al., 2020; Tseng et al., 2022).

In the analyzed articles, affective aspects and self-regulation in chemistry learning and laboratory were evaluated through questionnaires, interviews, and assessment checklists. These instruments provide information about students’ motivation, confidence, and ability to self-regulate the learning process. Affective experiences in the laboratory, for example, are measured through questionnaires that assess how students respond to experimental challenges, both emotionally and motivationally. This is important to create a learning environment that supports holistic student engagement in learning and laboratory. Research shows that affective aspects play a role in increasing student engagement and impact on improving student learning outcomes ( Nguyen et al., 2024; Shepard et al., 2018; Zheng et al., 2023).

### 3.3 RQ3: What are the emerging trends and key challenges in general chemistry assessment practices?

Recent studies indicate that the latest trend in general chemistry assessment is the increasing integration of technology-based assessment and the digitization of assessment practices in both lecture and laboratory contexts. Significant developments began during the COVID-19 pandemic in 2020, which accelerated the adoption of digital assessment technologies, including digital question banks, machine learning-assisted assessment systems, LMS-based assessments, and game-based quizzes. By 2024, more advanced technologies, such as virtual laboratory simulations, had become increasingly common, reflecting a major shift toward digital assessment practices in chemistry education.

Technology integration in assessment has been implemented in various forms, particularly in tests and assignments. Digital assessment tools include machine learning-assisted diagnostic tests, LMS-based assessments, quiz-based games, and instruments such as the Meaningful Learning in the Laboratory Inventory (MLLI). In assignment-based assessment, cloud-supported technologies such as smart worksheets, concept maps, online laboratory reports, and collaborative reflective notes using platforms such as Google Docs have become increasingly utilized in general chemistry laboratory assessment.

Several studies also demonstrated that technology-enhanced assessment practices improve assessment efficiency, provide real-time feedback, and support students’ self-reflection and independent learning ( Beerepoot, 2023; Muteti et al., 2022; Shepherd & Garrett-Roe, 2024). In addition, electronic question banks and flexible online assessment systems were found to support active learning by enabling students to access practice materials both inside and outside the classroom, thereby improving conceptual understanding, learning engagement, and learner autonomy ( Hagos & Andargie, 2024; Ling et al., 2024). The review also revealed that several assessment practices have begun integrating conceptual understanding with 21st-century skills, including scientific argumentation, metacognition, causal reasoning, experimental data analysis, and experimental design skills. Alternative assessment approaches, such as multidimensional rubrics, inquiry-based assessment, and reflective notes, were reported to improve not only students’ conceptual understanding but also their scientific argumentation and reflective thinking skills. In laboratory contexts, inquiry-based assessments remain widely used approaches because they provide opportunities for students to engage in authentic scientific investigations, ranging from experimental design to data analysis and scientific argument construction.

These findings indicate that future research will likely continue expanding the use of technology-enhanced assessment to support real-time feedback and multidimensional skill assessment. In developed countries, future assessment research may increasingly focus on advanced technologies such as augmented reality (AR), virtual reality (VR), artificial intelligence, and immersive laboratory assessment systems. Meanwhile, in developing countries, research is more likely to prioritize affordable, accessible, and contextually relevant technologies, including mobile-based applications, cloud-supported formative assessment systems, and low-cost digital platforms.

Nevertheless, several challenges remain. Limited internet access, inadequate educational infrastructure, budget constraints, data security concerns, and the need to align educational regulations with rapidly evolving assessment technologies continue to hinder the broader implementation of digital assessment practices. Furthermore, the growing diversity of assessment tools and technologies requires additional professional development and training for educators to ensure the effective implementation of technology-enhanced assessment strategies in general chemistry education.

## 4. Conclusion

This systematic literature review synthesized studies published between 2020 and 2024 on assessment practices in general chemistry learning and laboratory contexts. The findings indicate that assessment practices in general chemistry have undergone significant transformation through the integration of technology-based innovations, including machine learning-assisted assessment, digital assessment platforms, automated feedback systems, and game-based assessments. These innovations have contributed to improving assessment efficiency, flexibility, and student engagement in the learning process.

Despite these developments, conventional assessment approaches such as written examinations, laboratory reports, assignments, and direct observation remain dominant in general chemistry education. The review also revealed that various assessment instruments have been employed to evaluate diverse cognitive, metacognitive, scientific practice, and affective learning outcomes.

Furthermore, several important gaps remain in the literature. Research on assessment in general chemistry is still predominantly focused on theoretical learning rather than laboratory assessment or the integration of both domains. Studies integrating formative and summative assessment are still limited, while assessment practices explicitly designed to evaluate 21st-century skills, such as critical thinking, collaboration, creativity, communication, and problem-solving, remain underexplored.

Overall, this review contributes to the growing literature on chemistry education assessment by synthesizing current trends, innovations, and challenges in general chemistry assessment practices. The findings highlight the urgent need for more integrated, technology-enhanced, and competency-oriented assessment approaches that address both theoretical and laboratory learning in general chemistry education. In addition, this review provides evidence-based insights for educators, researchers, and policymakers in developing more innovative, inclusive, and effective assessment strategies to support improved learning outcomes in general chemistry education.

## 5. Limitations and future research

Several limitations of this review should be acknowledged. First, the review only included articles indexed in the Scopus database published between 2020 and 2024, which may have excluded relevant studies from other databases or earlier periods. Second, the review focused exclusively on English-language publications, potentially overlooking important contributions published in other languages. Third, only open-access articles were included, which may have limited the inclusion of potentially relevant studies published in subscription-based journals. In addition, this review employed a narrative synthesis approach without conducting a quantitative meta-analysis. Consequently, the effectiveness of different assessment methods and technologies could not be statistically compared. Formal risk-of-bias assessment was also not conducted due to the heterogeneous nature of the included studies, which consisted of diverse research designs, assessment contexts, and outcome measures. Although all included articles originated from peer-reviewed and Scopus-indexed journals, the absence of a standardized quality appraisal procedure remains a methodological limitation of this review. Another limitation concerns the geographical distribution of the reviewed studies. Most studies originated from developed countries, particularly the United States and several European and Asian countries with advanced educational infrastructures and technological resources. Therefore, the findings may not fully represent assessment practices and challenges in developing countries with different educational conditions, technological access, and institutional capacities.

Future research should further investigate the integration of formative and summative assessment within both theoretical and laboratory-based general chemistry learning. More comprehensive assessment models are needed to evaluate not only conceptual understanding but also practical competencies and 21st-century skills, including critical thinking, collaboration, creativity, scientific communication, and problem-solving abilities. Further studies are also recommended to explore the effectiveness of emerging technologies such as artificial intelligence (AI), adaptive assessment systems, augmented reality (AR), virtual reality (VR), learning analytics, and automated feedback systems in supporting chemistry assessment practices. Comparative and quantitative studies, including meta-analyses, are necessary to evaluate the relative effectiveness of different assessment strategies and technological innovations across diverse educational contexts. Considering the disparities between developed and developing countries identified in this review, future research should prioritize the development of affordable, accessible, and contextually relevant assessment technologies suitable for resource-constrained educational environments. Mobile-based assessments, cloud-supported formative assessment systems, and low-cost digital platforms may provide promising alternatives for improving assessment accessibility in developing regions. Finally, greater international collaboration among researchers, educators, institutions, and policymakers is needed to reduce disparities in assessment innovation and implementation. Such collaboration may support the sharing of expertise, development of educational infrastructure, educator professional training, and the establishment of more inclusive and equitable assessment practices in general chemistry education.

## Data Availability

The PRISMA 2020 checklist, PRISMA flow diagram, and the dataset underlying this systematic literature review (including the study extraction table) are openly available in the Open Science Framework (OSF) repository under a Creative Commons Zero (CC0 1.0 Universal) public domain dedication. The repository record is accessible at https://doi.org/10.17605/OSF.IO/HSMEA ( Oktariani et al., 2026). The repository contains the following materials:
1. Dataset SLR Data Sharing.xlsx 2. PRISMA 2020 Flow Diagram of the Study Selection Process.png 3. PRISMA 2020 Checklist.pdf 4.
Tables and Figures Formal citation: Oktariani, Nahadi, Munawaroh, H. S. H., Rusyati, L., Baruri, A., Mellyzar, Almubarak, Nabuasa, D. A. (2026). Systematic Review of Assessment in General Chemistry: Trends, Digital Innovations, and Integration of Theory-Laboratory Course. Open Science Framework (OSF). https://doi.org/10.17605/OSF.IO/HSMEA Licensed under CC0 1.0 Universal.
