# Supplementary material for: Systematic Review of Assessment in General Chemistry: Trends, Digital Innovations, and Integration of Theory-Laboratory Course
Source: F1000Res. 2026 Aug 8;15:1338. [Version 1] doi: 10.12688/f1000research.182947.1 (PMC13627939; doi:10.12688/f1000research.182947.1)
Supplement: Peer review history

# Reviewer report: Reviewer response for version 1

**DOI:** 10.5256/f1000research.201934.r524913

**Published:** 2026-09-30

**Authors:** Achmad Rante Suparman

**Affiliations:** 1 Universitas Papua, Manokwari, Indonesia

The methodology is well-structured, adhering to the PRISMA framework; however, in Figure 1 (PRISMA Flow Diagram), the "Records excluded" box following the title and abstract screening indicates n=52. This leaves 52 articles (107 minus 55; "Reports sought for retrieval"). Yet, in the subsequent box ("Reports assessed for eligibility"), the count suddenly shifts to n=30 without an explicit explanation in the "Reports not retrieved" box (listed as n=0). Please correct the numerical flow in the PRISMA diagram boxes to ensure mathematical consistency (107 minus 55 abstracts = 52 full-text articles; 52 minus 22 full-text exclusions = 30 selected articles).

 The authors relied solely on Scopus as the search source. This approach potentially excludes significant literature indexed in Web of Science (WoS) and ERIC. Please explain this in the Discussion section.

 Excluding 280 articles simply because they are not open access risks introducing publication or selection bias. Many high-quality studies on chemistry laboratory evaluation published in subscription-based journals (such as the ACS Journal of Chemical Education) might be overlooked in the analysis if they are not open access. Please address this point in the Discussion section.

Are the rationale for, and objectives of, the Systematic Review clearly stated?

Yes

Is the statistical analysis and its interpretation appropriate?

Yes

If this is a Living Systematic Review, is the ‘living’ method appropriate and is the search schedule clearly defined and justified? (‘Living Systematic Review’ or a variation of this term should be included in the title.)

Yes

Are sufficient details of the methods and analysis provided to allow replication by others?

Partly

Are the conclusions drawn adequately supported by the results presented in the review?

Yes

Reviewer Expertise:

Chemistry Education, Chemistry Education Assessment, Educational Measurement, Psychometrics, Instrument Development and Validation, and AI-Assisted Scientific Inquiry in Chemistry Education.

I confirm that I have read this submission and believe that I have an appropriate level of expertise to confirm that it is of an acceptable scientific standard.

# Reviewer report: Reviewer response for version 1

**DOI:** 10.5256/f1000research.201934.r510159

**Published:** 2026-09-16

**Authors:** Anggun Zuhaida

**Affiliations:** 1 Universitas Islam Negeri Salatiga, Salatiga, Central Java, Indonesia

The SLR provides a detailed, systematic, and well-structured review process. However, the discussion does not yet provide sufficiently strong recommendations to extend the broader impact and contribution of the findings to the development of assessment practices and research in chemistry education. Therefore, it is recommended that the authors add research questions that further investigate the competencies and learning aspects targeted by assessment in general chemistry and laboratory education, as well as the research gaps in the development and implementation of chemistry assessment practices. Addressing these two aspects would strengthen the synthesis of the findings and enable the review to provide more meaningful recommendations and directions for future research, thereby enhancing the overall contribution and broader impact of the article.

Are the rationale for, and objectives of, the Systematic Review clearly stated?

Yes

Is the statistical analysis and its interpretation appropriate?

Partly

If this is a Living Systematic Review, is the ‘living’ method appropriate and is the search schedule clearly defined and justified? (‘Living Systematic Review’ or a variation of this term should be included in the title.)

Yes

Are sufficient details of the methods and analysis provided to allow replication by others?

Yes

Are the conclusions drawn adequately supported by the results presented in the review?

Yes

Reviewer Expertise:

The SLR provides a detailed, systematic, and well-structured review process. However, the discussion does not yet provide sufficiently strong recommendations to extend the broader impact and contribution of the findings to the development of assessment practices and research in chemistry education. Therefore, it is recommended that the authors add research questions that further investigate the competencies and learning aspects targeted by assessment in general chemistry and laboratory education, as well as the research gaps in the development and implementation of chemistry assessment practices. Addressing these two aspects would strengthen the synthesis of the findings and enable the review to provide more meaningful recommendations and directions for future research, thereby enhancing the overall contribution and broader impact of the article.

I confirm that I have read this submission and believe that I have an appropriate level of expertise to confirm that it is of an acceptable scientific standard.
